# PTEN, Longevity and Age-Related Diseases

**DOI:** 10.3390/biomedicines1010017

**Published:** 2013-12-13

**Authors:** Izak S. Tait, Yan Li, Jun Lu

**Affiliations:** 1 School of Applied Sciences, Auckland University of Technology, WS306, WS Building, City Campus, St Paul Street, Auckland 1010, New Zealand; E-Mails: zacktait@gmail.com (I.S.T.); yan.li@aut.ac.nz (Y.L.); 2 School of Interprofessional Health Studies, Auckland University of Technology, AF Building, North Shore Campus, 90 Akoranga Drive, Auckland 0627, New Zealand; 3 Institute for Applied Ecology New Zealand, Auckland University of Technology, WL Building, City Campus, St Paul Street, Auckland 1010, New Zealand; 4 Institute of Biomedical Technology, Auckland University of Technology, WD Building, City Campus, St Paul Street, Auckland 1010, New Zealand

**Keywords:** PTEN, aging, longevity, caloric, restriction, DNA, damage, gene

## Abstract

Since the discovery of PTEN, this protein has been shown to be an effective suppressor of cancer and a contributor to longevity. This report will review, in depth, the associations between PTEN and other molecules, its mutations and regulations in order to present how PTEN can be used to increase longevity. This report will collect recent research of PTEN and use this to discuss PTEN’s role in caloric restriction, antioxidative defense of DNA-damage and the role it plays in suppressing tumors. The report will also discuss that variety of ways that PTEN can be compromised, through mutations, complete loss of alleles and its main antagonist, the PI3K/AKT pathway.

## 1. Introduction

Phosphatase and tensin homolog deleted on chromosome 10 (PTEN, also known as MMAC1 and TEP1) was first discovered in 1997 by two independent groups and recognized as the long sought after tumor suppressor gene frequently lost on human chromosome 10q23 [1,2]. This locus is highly susceptible to mutation in human cancers: the frequency of mutations have been estimated to be 50%–80% in sporadic tumors such as glioblastomas, prostate cancers and endometrial carcinomas; and 30%–50% in lung, colon and breast tumors. PTEN is often associated with advanced cancers and metastases [3], due to loss of PTEN having been observed at its highest frequency in late stages of cancers. 

Together with p53, Ink4a and Arf, PTEN makes up the four most important tumor suppressors in mammals [4] as evidenced by their overall high frequency of inactivation across a variety of cancer types. Because of this, it is vital to understand the mechanisms of how PTEN functions.

The gene that encodes PTEN is non-redundant and expressed in all eukaryotic cells [5]. While there is only one homologue of PTEN found in fungi and lower animals, several orthologues have been discovered in mammals, namely TPTE, PTEN2 and TPIP [6,7,8]. Unlike PTEN, however, these orthologues are not ubiquitously expressed in all tissues. TPTE and PTEN2 are only expressed in the testis, while TPIP is expressed in the testis, brain and stomach. Whilst the orthologues share PTEN’s major function, that of phosphatidylinositol (3,4,5)-triphosphate (PIP3) phosphatase (as will be discussed later), analyses have shown that the orthologues are limited to the Golgi apparatus and endoplasmic reticulum, while PTEN is expressed in the nucleus, cytoplasm and at the cellular membrane. The difference in localization demonstrates that the PTEN orthologues do not regulate PIP3 in the same manner as PTEN, reinforcing the non-redundancy of PTEN.

The PTEN protein contains 403 amino acids and several domains [1]. The crystal structure of PTEN shows a 179 residue *N*-terminal domain and a 166 residue *C*-terminal domain [9]. The *N*-terminal domain contains a protein tyrosine phosphatase (PTP) signature motif that is similar to dual specificity protein phosphatases. The *C*-terminal domain contains the C2-domain that is responsible for its recruitment to phospholipid membranes. The PTEN homologue found in fungi and lower animals lack this C2 domain [1]. 

While the functions of PTEN will be discussed in depth in the report, in brief the main function of PTEN is to antagonize the PI3K/AKT pathway [10], thereby opposing the pathway’s cell proliferative response and, more important to longevity, opposing AKT’s downregulation of antioxidant genes and proteins [11]. In concert with this function, PTEN has been reported to bind with another antioxidant gene, *p53*, and arresting the cell cycle whilst positively regulating protein dealing with DNA-damage [12]. These functions serve not only to extend cellular longevity but also prevent deleterious DNA-damage that can lead to malignant tumors [13]. 

The purpose of the report is to serve as a comprehensive review of the links that have been made between PTEN and the potential effects it may have on ageing. It will cover various issues such the regulators of PTEN, the regulatory effects of PTEN, its cellular functions, its associations with cancer and its direct effects on longevity in the effort to understand the many and varied pathways that PTEN is a part of, and how these intricate and integral pathways are key to effecting longevity. While Ponce de León’s dream of a fountain of youth may be unobtainable as of yet, this report will show that extended longevity is highly possible.

This paper follows in theme on recent papers such as Jaskelioff *et al.* [14] and Ortega-Molina *et al.*, which show the strides that anti-ageing research have made over recent years [4,14,15]. PTEN has the potential the play a crucial role alongside these other studies as, beyond its documented ability to extend longevity, its function as a tumor repressor is vital to any lasting extended longevity to prevent the rise of tumors often associated with extended longevity.

With recent and current studies already being done on PTEN’s effect on longevity, it is necessary to comprehend the basics of PTEN to be able to understand its role in the myriad pathways of the human cell. These pathways are interconnected and intertwined with many feedback loops, and finding the areas which can be exploited for the betterment of human ageing, whilst at the same time not compromising others to our detriment is a very exhaustive process and running a fine line. This is the reason for this report, to understand those fundamentals to be able to understand the grander picture.

## 2. PTEN Regulations

### 2.1. Regulates PTEN

#### 2.1.1. Mutations

Of the varied sources that can act as regulators upon PTEN, mutations can have a much more significant impact on the structure and function of PTEN as it appears before transcription and translation have occurred. PTEN haploinsufficiency (where only a single functional allele remains) have been shown to contribute to tumor progression and even minor deficiency of function can aid tumor development [16]. This is evident in many cancers and syndromes (to be discussed in depth further on) such as Cowden syndrome, whose C2-domain mutations may retain partial or full PTEN lipid phosphatase functionality, as seen in biochemical assays [17,18]. By truncating phosphorylated residues on the *C*-terminal, mutations such as these can affect PTEN stability, phosphorylation, protein interactions and proper localization [19]. Mutation of Lys289 has also been found to alter PTEN localization.

The Ser380, Thr382 and Thr383 cluster on the C-terminal (referred to as the STT [20]) have been shown to significantly negatively affect PTEN stability while positively affecting phosphatase activity when mutated [21], despite the fact that mutation of the major sites for phosphorylation, Ser370 and Ser385, have been shown to have little effect on PTEN function [22]. Leslie and Downes suggests STT phosphorylation renders PTEN into a “closed” state while mutation renders it “open”, increasing PTEN’s interaction with binding partners, making it more unstable by rendering it more susceptible to proteolysis [22]. Because of this, it has been proposed that PTEN’s basal state is that of a phosphorylated, inactive state, being activated by dephosphorylation of the STT cluster to foster conformational changes. Further strengthening this hypothesis is the fact that tumor-derived *C*-terminal mutants of PTEN are highly susceptible to proteolysis, indicating the *C*-terminus to be responsible for protein stability [23].

Mutations on the *N*-terminal are also thought to affect the stability of the PTEN protein whilst not inactivating phosphatase activity [17].

The evidence of mutational regulation of PTEN is further supported by the PTEN hamartoma tumor syndromes (PHTS) [24] a group of autosomal dominant syndromes identified by developmental disorders, hamartomas, neurological deficiencies and an increased risk of cancer. This shows that loss, or mutation, of an allele directly leads to a deficiency of PTEN function thereby resulting in PHTS and an increased cancer risk.

#### 2.1.2. Transcriptional Regulation

Despite the fact that the exact nature of PTEN regulation is still unclear, there are a number of factors that have been demonstrated to upregulate PTEN transcription. One of these is the transcription factor peroxisome proliferation-activated receptor γ (PPARγ), a regulator of glucose metabolism and fatty acid storage. PPARγ upregulates PTEN via its selective ligand rosiglitazone on peroxisome proliferator response elements (PPRE) identified on the PTEN promoter region [25]. PPARγ is itself regulated by oleamide, a fatty acid primary amide that has been implicated in sleep induction and hypolocomotion [26], giving an indication of the complex network that surrounds PTEN. In addition, ligand-activated PPARδ up-regulates PTEN and suppresses the phosphatidylinositol 3-kinase (PI3K)/AKT pathway [27]. Knockdown of PTEN with siRNA abrogated the effects of PPARδ on cellular senescence and on generation of ROS in angiotensin II treated vascular smooth muscle cells. 

The early growth-regulated transcription factor-1 (EGR-1) upregulates PTEN messenger RNA, and thus protein, expression leading to increased levels to apoptosis (a function of PTEN) [28]. It accomplishes this by binding to a functional GCGGCGGCG Egr-1-binding site on an untranslated region on the 5' strand. Much like PTEN, EGR-1 is involved in growth restricting and apoptotic processes [29] and it has been hypothesized that the interactivity of both proteins is a result of the similarity of their functions, that part of PTEN’s apoptotic function is due to EGR-1. This is supported by Virolle *et al*.’s experiment which showed that the introduction of exogenous EGR-1 restored PTEN stimulation in EGR-1^−/−^ cells *in vitro*.

EGR-1 can also be stimulated by IGF-2 in a negative feedback loop [30]. According to Moorehead *et al*.’s study, IGF-2 regulates and is regulated by EGR-1, and while EGR-1 stimulates PTEN and IGF-2 stimulates the AKT pathway [31] (two opposing pathways). Introduction of IGF-2 has been found to increase AKT levels in the short term while increasing PTEN levels in the long term. This is suggestive of a negative feedback loop to counter the cell-proliferative properties of the AKT pathway.

Ras, a small GTPase, regulates the passage of extracellular signals to intracellular pathways by acting as a molecular switch [32]. Ras can act as an antiapoptotic/cell-survivalist regulator by inducing the RAS-RAF-MEK-ERK pathway, down-regulating PTEN via the transcriptional factor c-Jun [33], and inducing the AKT-PI3K-NF-kappaB pathway, down-regulating *p53* and *FoxO* genes [34]. It is unsurprising, therefore, to discover that Ras is a key oncogenic protein.

Epigenetic effects may inhibit PTEN expression [35] such as promoter hypermethylation in various types of cancer [36,37,38]. Caution must be taken when interpreting epigenetic silencing regarding PTEN as a PTEN-pseudogene exists with a promoter also shown to be methylated [39] although there is doubt about the expression of the pseudo-gene [40].

PTEN is negatively affected by MicroRNAs (miRNAs) which are short, single-stranded endogenous RNAs approximately 22 nucleotides in length that repress mRNA translation. Specifically, it was shown that PTEN is inhibited by miR-21 [41,42], one of the most frequently found miRNAs to be upregulated in cancer to promote cell proliferation and to inhibit apoptosis [43,44,45]. This suggests that its oncogenic effect is due, at least in part, to its suppression of PTEN. The oncogenic effects of PTEN inhibition will be discussed in detail later on.

#### 2.1.3. Post-Translational Regulation

By far the greatest number of regulatory effects on PTEN occurs post-translation, by the interaction of other proteins and chemicals on the PTEN protein. The various post-translation modifications that may regulate PTEN include phosphorylation, acetylation, oxidation and ubiquitination. 

For example, it has been reported that PTEN stability is subject to various post-translational modifications such as phosphorylation of specific residues on its *C*-terminal tail, as done by protein interacting with carboxyl-terminus tail 1 (PICT1) for instance [46], that have been associated with increased stability [21,47,48] while phosphorylation of other sites such as Thr366 destabilize PTEN [49]. A total of six phosphorylation sites, at Thr366, Ser370, Ser380, Thr382, Thr383, and Ser385, have been shown to regulate PTEN tumor suppressing function (by modulating protein stability). Phosphorylation also results in decreased catalytic activity toward lipid substrates, resulting in a decreased ability to interact with membranes, a key function of PTEN.

Depletion of serine/threonine-protein kinase (Chk1), a signal transducer in the cell cycle pathway [50], decreases phosphorylation and levels of PTEN. Chk1 and PTEN are linked in the cell cycle regulatory pathway as phosphorylation of both Chk1 and PTEN recovers the cell cycle after DNA replication has stalled. An ATR-Chk1-CK2-PTEN pathway also exists as ATR phosphorylates Chk1 at Ser137, which in turn induces casein kinase 2 (CK2)-mediated phosphorylation of PTEN at Thr383 [48]. This is vital for recovery of the cell cycle and illustrates the roles of Chk1 and PTEN in DNA damage response.

Rho-associated protein kinase (ROCK), a protein involved in cellular membrane activities, has been shown to increase PTEN localization to the plasma membrane by phosphorylating sites Ser229, Thr322, Thr319, and Thr321 in the C2 domain, in contrast to the above mentioned phosphorylation of site Thr366 [51]. Phosphorylation of PTEN has also been attributed to glycogen synthase kinase 3β (GSK3β) [52,53].

PTEN contains two PEST-sequence (proline, glutamic acid, serine, threonine) motifs which are normally found on short-lived, unstable proteins which are degraded by ubiquitin mediation of the proteasome. While PTEN is, under normal circumstances, a stable and long lived protein, studies have shown that PTEN may be regulated by the PEST-sequence domains in that the half-life of PTEN is increased during proteasome inhibition [48]. It was also shown that exposure to zinc-ions initiated ubiquitin-dependent, proteasomal degradation of PTEN [54]. Wu *et al*. found that proteasome inhibition prevented PTEN degradation in response to zinc-ions, suggesting that zinc-ions activate the degradation process either through the proteasome or through ubiquitin.

In contrast to this, however, Tang and Eng found that p53 may destabilize and degrade PTEN in cells with proteasome dysfunction [55].

Lys13 and Lys289 are two conserved sites identified for PTEN ubiquitination and Trotman *et al*. showed that ubiquitin conjugation to these sites is highly important for the shuttling of PTEN between nucleus and cytoplasm [19]. Wang *et al*. isolated E3 ubiquitin-protein ligase 4-1 (NEDD4-1) and showed that overexpression of NEDD4-1 mediated mono- and poly-ubiquitination of PTEN through physical interaction [56].

The nuclear histone acetyltransferase-associated PCAF protein promotes PTEN acetylation at Lys125 and Lys128 during PTEN interaction to decrease the catalytic activity of PTEN [57]. The catalytic activity can also be downregulated by oxidation of reactive oxygen species (ROS) which cause the formation of a disulfide bond between Cys124 and Cys71 [58]. This has been seen to either take the form of hydrogen peroxide (H_2_O_2_) or endogenous ROS produced in macrophages in response to cellular and metabolic stress and is associated with oxidant-dependent downstream signaling [59,60]. 

Proteins such as Na^+^–H^+^ exchanger regulatory factor (NHERF) as well as membrane-associated guanylate kinase inverted 2 (MAGI-2) can regulate PTEN localization and recruitment to the membrane through the 3 aa *C*-terminal region on PTEN [47,61]. These PDZ domain interactions can be negatively modulated by phosphorylating PTEN on its *C* terminus [21,23,62]; however, deletion of the three aa amino acids does not alter the tumor suppressive activity of PTEN [47]. Table 1 below is a brief summary of regulators of PTEN mentioned in this section.

**Table 1:** Regulators of PTEN and their effects.

PTEN regulators	Effects
Haploinsuffiency	Reduced PTEN function, higher susceptibility to tumours [16]
C2-domain mutations	Negatively affect PTEN stability, phosphorylation, protein interactions and proper localization [17,18,19]
*N*-terminal mutations	Negatively affect protein stability [17]
PPARγ	Up-regulation of PTEN transcription [25]
EGR-1	Up-regulation of PTEN mRNA leading to apoptosis [28]
Ras	Down-regulates PTEn via c-Jun, induces PI3k-AKT pathway [33]
Promoter hypermethylation	Possible inhibition of PTEN expression [35]
STT phosphorylation	Significantly negatively affect PTEN stability while positively affecting phosphatase activity [21,22]
mir-21	Down-regulates PTEN transcription [41,42,43,44,45]
PICT1	Phosphorylates PTEN *C*-terminal, increases stability [21,47,48]
Phosphorylation of T366, S370, S380, T382-3, S385	Affects protein stability and decreases PTEN’s ability to interact with membranes [49]
Chk1	Induces CK2-mediated phosphorylation of PTEN, recovers stalled cell cycle [48,50]
ROCK	Phosphorylates PTEN to increase PTEN localization to membrane [51]
PEST-sequences	Destabilizes PTEN [48]
NEDD4-1	Ubiquitinates PTEN via L13, L289 [19,20,21,22,23,24,25,26,27,28,29,30,31,32,33,34,35,36,37,38,39,40,41,42,43,44,45,46,47,48,49,50,51,52,53,54,55,56]
PCAF	Promotes PTEN acetylation to decrease PTEN catalytic activity [57]
ROS	Decreases PTEN catalytic activity [58]
NHERF & MAGI-2	Regulates PTEN localization and recruitment to the membrane [47,61]
NFκB	Down-regulates PTEN transcription [63]

### 2.2. Regulated by PTEN

#### 2.2.1. In the Cytoplasm

Of the various molecules that are regulated by PTEN, the most well documented is the phosphatidylinositol (3,4,5)-trisphosphate [PtdIns(3,4,5)P3]/Protein Kinase B pathway, otherwise referenced as the PIP3/AKT pathway, which PTEN antagonizes [10,64]. PTEN dephosphorylates PIP3 by removing the D3 phosphate from the inositol ring, resulting in PIP2 [10]. PIP3 is responsible for the recruitment of proteins containing pleckstrin homology domains to the cellular membranes, including the AKT isoforms and PDK1 as evidenced upon PTEN inhibition. The AKT pathway promotes cell survival and proliferation as will be discussed further on.

A study done by Vivanco *et al*. showed that the Jun-*N*-terminal Kinase (JNK) pathway to be activated upon PTEN loss, suggesting it is down-regulated by PTEN [65]. Further investigation led the authors to discover this was through the Phosphatidylinositide 3-kinase (PI3K) family of proteins of which PIP3 is a part. While PIP3, as mentioned above, is a well-known regulator of AKT, the activation of JNK was AKT independent. The exact process how PIP3 activates JNK is as yet unclear [63]. Xia *et al.* showed how JNK, through its upstream protein mitogen-activated protein kinase kinase-4 (MEKK4), down-regulates the transcription of PTEN by activating the transcription factor NFκB whereby it binds to the promoter sequence of PTEN [63]. This suggests a negative feedback loop between PTEN and JNK.

#### 2.2.2. In the Nucleus

A p53 binding sequence has been identified on the promoter sequence of PTEN and a survival mechanism that only functions through the transcription of PTEN [66]. PTEN has also been reported to bind to p53’s *C*-terminus directly via its C2 domain, increasing its stability and transcription and therefore increasing p53 protein levels. This is further demonstrated by a study showing the drastically reduced half-life of p53 in PTEN-null mouse cells [67] independently of PTEN phosphatase activity, suggesting it is the binding of PTEN that gives this result. Contrasting studies have shown, however, a marked up-regulation of p53 in the absence of PTEN [68]. In addition, PTEN-loss induced senescence is associated with enhanced p53 translation [69].

Li *et al.* showed another method how PTEN can influence p53: by the interaction of PTEN with the transcriptional coactivator p300/CBP (CREB-binding protein) [70]. By forming a complex with PTEN, p300 acetylates p53 on sites Lys373 and Lys382. This results in stabilization and tetramerisation of p53. This in turn allows PTEN to bind to p53, and described above, further stabilising the p53–p300 complex allowing a more efficient acetylation of p53. The net result in all of this is maximum activation of p53 DNA binding and transcription. Li *et al.* [70] also showed an increase in this process during radiation, implying this process is in response to DNA damage.

It has been suggested that PTEN has a role in maintaining chromosomal integrity by extensive centromere breakage and chromosomal translocations observed in PTEN null cells [71,72]. Puc and Parsons showed this to be due to AKT-phosphorylation of CHK1 that leads to sequestration of CHK1 from the nucleus and, as explained above, induces phosphorylation and levels of PTEN. The regulation of centromere stability is done by PTEN physically associating with the centromere binding protein Centromere Protein C (CENP-C) [72]. This is done in a phosphatase independent manner but requires a functional *C*-terminus on PTEN’s behalf. Shen *et al.* also showed that PTEN positively regulates Rad51, a protein involved in repairing double stranded breaks, through the transcription factor E2F-1 [72]. Table 2 below is a brief summary of the regulatory effects of PTEN.

**Table 2:** The regulatory effects of PTEN.

Regulated by PTEN	Effects
PIP3/AKT pathway	Increased longevity [73,74,75,76], reduced insulin signalling [77], tumour suppression [78], reduced DNA damage [79]
JNK pathway	Downregulated via phosphorylation PI3K [65]
p53	Increased stability and transcription of p53 [67]
CENP-C	Binds with PTEN, maintains chromosomal integrity [72]
Rad51	Positively regulated via E2F-1, repairs DSB [72]

## 3. Cell Functions

**Figure 1:**
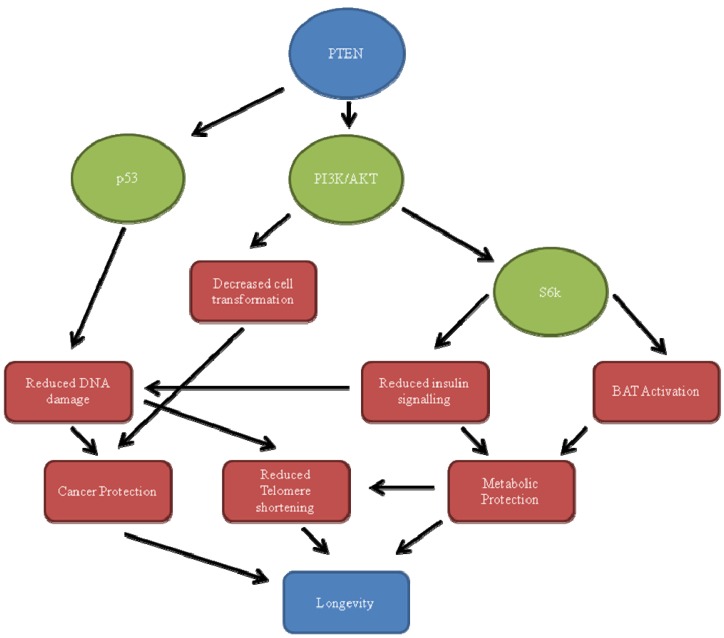
Mechanisms connecting PTEN with longevity. The major functions of PTEN can be seen here: Through down-regulation of the PI3K/AKT pathway (and thus S6K), there is a reduction in cellular transformation, insulin signaling and BAT activation resulting in cancer protection, metabolic protection and reduced telomere shortening. By up-regulating p53, DNA damage is reduced as well as telomere shortening. The result of these is an increased longevity.

The regulatory effects of PTEN are synonymous with its cellular functions as PTEN functions by affecting a variety of pathways. While the report above focused on the mechanics of PTEN regulations, the report below will focus on the effects of this and how it relates to longevity. In Figure 1, one can see the major functions of PTEN mapped out and how they relate to longevity. It is important to note that, while this section of the report deals with each variable individually, one can see in Figure 1 that it is only through a handful of interactions that PTEN has the potential to increase longevity. This is because of the crucial link that the PI3K/AKT pathway have within the cellular system as will be discussed below.

### 3.1. PI3K

The most prominently reported function of PTEN is to antagonize the PI3K signaling pathway [10,64,80]. PI3Ks can be stimulated by growth stimuli or insulin stimuli [4], whereupon class proteins I of the PI3K family such as PIP2 are catalyzed to convert to PIP3, a second messenger protein that stimulates growth, proliferation, survival and inhibition of apoptosis [11,81]. PIP3 is responsible for recruiting proteins containing a pleckstrin homology domain to cellular membranes, including the AKT isoforms. Once there, AKT isoforms are phosphorylated at two residues: on Thr308 by PDK1 [82] and Ser473 by mTOR kinase complex 2 (mTORC2) [83] which is required for full activation. As such, termination of AKT activity is brought about by dephsophorylating AKT isoforms on Ser473 [84,85].

AKT is responsible for phosphorylating many cellular substrates to promote survival, growth, proliferation and metabolism such as mouse double minute 2 homolog (MDM2), glycogen synthase kinase 3 (GSK3), forkhead transcription factors (FOXO), Bcl-2-associated death promoter (BAD), Caspace-9, and p27 [86] as well as activating mammalian target of rapamycin complex 1 (mTORC1) through phosphorylating the TSC tumor suppressor complex and activation of Ras-homolog-enriched-in-brain (RHEB), a Ras-related GTPase [87]. There are several feedback loops incorporated into this cascade, one negative feedback loop being mTORC1 which works to inhibit AKT activation [88] as well as a positive feedback loop, the inhibition of FOXO transcription factors by AKT which, when activated, suppresses insulin signaling to initiate the PI3K/AKT cascade [77,89]. 

A significant role of the PI3K/AKT cascade is to inhibit apoptosis. One method is by phosphorylating pro-caspase 9 on residues Ser196 and Thr125 [90]. Dephosphorylated pro-caspase 9 binds to, cleaves and activates caspases 3 and 7, which target key regulatory and structural proteins for proteolysis, resulting in cell death [91]. Another method is by phosphorylating Mdm2, resulting in its nuclear localization leading to the subsequent export and degradation of p53 [92,93], a key aspect of PTEN’s pathways as mentioned earlier.

Another role of the AKT pathway is the dual regulation of the forkhead transcription factors FOXO and the NF-κB, the former involved in longevity and the latter in causing the ageing process [73,74,75,76]. By phosphorylating the FOXO proteins, AKT ensures their retention in the cytosol in a complex with the 14-3-3 protein. With reduced insulin signaling, enacted by various methods including a negative feedback induced by FOXO, FOXO proteins translocate to the nucleus whereby they form complexes with co-activators [94,95,96]. These complexes then allow FOXO to induce the expression of several antioxidative enzymes, stress resistance inducers [94,97], regulation of immunosenescence [98] and oxidative stress. Conversely, NF-κB is responsible for down-regulating FOXO and antioxidative proteins [74,75,76].

The importance to longevity here is twofold. Firstly, by inhibiting the PI3K pathway, FOXO proteins are indirectly overexpressed leading to an increased antioxidant, stress resistant and immune activity. The enhanced antioxidant levels would result in a reduction of DNA-damage and telomere damage, for example, the single strand and double strand break lesion induced by reactive oxygen species (ROS) [79], whilst the increased stress resistance and immune activity would result in an increased capability to fight off disease.

The second impact inhibition of the PI3K pathway can have on longevity is by the inhibition of its cell proliferative abilities [78]. Beyond the decreased risk of cancer that curtailing excessive cell proliferation would bring, apoptosis contains and disposes of cellular toxins that have the potential to damage tissues.

### 3.2. Membrane

Das *et al.* discovered, through the use of a fluorescent mutant version of PTEN, that PTEN is expressed at the plasma membrane [99]. This was in contrast to previous studies showing PTEN to be expressed only in the cytoplasm. More recent immunohistochemical studies have shown the distribution of PTEN various between tissues: in epithelial cells such as skin and colon, between is mostly found in the cytoplasm [100,101]; in neurons, fibroblasts, adrenal medulla and thyroid most PTEN is in the nucleus [102,103,104]; polarized MDCK cells show that PTEN localizes at the membrane in cell–cell tight junctions [61]. 

A possible explanation for the different localization patterns may involve the 50 amino, C2 domain, on the *C*-terminus of PTEN. As mentioned earlier, this region contains multiple residues for phosphorylation, such as by CK2 and also a PDZ-binding domain that can bind to MAGI2 [61,99], a protein localized at the cellular membrane. At the membrane, PTEN has been implicated to exert effects on the cytoskeleton and suppress cell migration [105]. This has been indicated by an increased migration in PTEN null embryonic fibroblasts. Elevated PIP3 in these cells activates Rac1 and Cdc42, small GTPase mediators of cellular migration [106]. Raftopoulou *et al.* showed that phosphorylation of the Thr383 residue on the C2 domain inhibited PTEN’s effect on migration [107].

### 3.3. Nucleus

#### 3.3.1. Localization

It was initially assumed that PTEN was strictly a cytoplasmic protein, due to the reported lipid-binding domain, an absence of a nuclear localization signal (NLS) on PTEN, and overexpression studies and PTEN antibodies that showed PTEN to be exclusively in the cytoplasm [9,108,109]. It is now known, however, to be present and functional in the nucleus [110].

More recent studies done with reliable PTEN antibodies have produced immunocytological and immunohistochemical data that demonstrates the presence of PTEN in primary, differentiated cells such as neurons [111], pancreatic cells [112], vascular smooth muscle cells [113], thyroid tissue [104], and in the intestinal mucosa [19]. Ginn-Pease and Eng showed that the concentration of nuclear PTEN mirrors that of cytoplasmic PTEN during the cycle [114]. The highest concentration of nuclear and cytoplasmic PTEN is during the G0–G1 phase when the cell is quiescent or undergoing protein synthesis. In contrast, rapidly cycling cell lines have shown a marked decrease in PTEN localization [111,114,115] indicating that the nuclear localization of PTEN may not only be dependent on cycle stage but also on differentiation status.

Mechanisms by which PTEN enters the nucleus include diffusion, active shuttling, cytoplasmic-localization-signal-dependent export and monoubiquitylation-dependent import [116]. PTEN has also been associated with the Major Vault Protein (MVP), a nuclear-cytoplasmic transport protein which was found to mediate PTEN nuclear importation. This is dependent on NLS-like residue sequences on PTEN: NLS4 (amino acids 65–269 KKDK), NLS2 (amino acids 60–164 RTRDKK) and NLS3 (amino acids 233–237 RREDK) [117]. NLS4 is necessary for PTEN importation but requires either NLS2 or NLS3 for proper function. These allow MVP to mediate PTEN and Chung *et al.* found this process to be independent of PTEN phosphorylation and of its phosphatase activity [117].

It was more recently reported that nuclear transportation of PTEN is cell cycle dependent and regulated by the PI3K/AKT/mTOR/S6K signaling cascade [118], specifically the export through S6K phosphorylation of PTEN and a CRM1-dependent mechanism mediating its export. Activation of S6K initiates a negative feedback loop by inhibiting IRS-1, slowing the PI3K/AKT cascade, but not stopping it due to activation by growth factors. As PTEN is implicated in cell cycle arrest, it has been suggested that this export is a method of controlling this arrest.

#### 3.3.2. Nuclear Interactions

PTEN has been implicated to play a role in a variety of nuclear functions such as chromosome stability, DNA repair, cellular stability and the aforementioned cell cycle arrest. Chang *et al.* found that oxidative stress retards the nuclear exportation of PTEN due to the dephosphorylation of Ser380, thus accumulating PTEN in the nucleus [12]. This, the authors found, allows PTEN to bind to p53 (as shown above) and enhance p53-mediated functions, which showed a decrease in cellular ROS in a p53-dependent manner and an increase in the p53 downstream antioxidant gene *Sestrin*. 

PTEN was also found to arrest the cell cycle via p53 during the G1 phase, suggesting that the cause of this was for DNA repair [12]. It was also found the PTEN could arrest the cell cycle at the G1 phase by suppressing the transcription of cyclin D1 through phosphorylation of the MAPK pathway [119] or by limiting its nuclear accumulation [120].

PTEN was shown to increase chromosomal stability by binding to CENP-C to associate with the centromere and by increasing the transcription of Rad51 to repair double-strand breaks, independent of its phosphatase functions [71,72]. The importance of stable and integral chromosomes cannot be understated. Poor chromosomal stability due to DSB has been found to lead to loss of heterozygosity leading to tumorogenesis (as reviewed in van Gent *et al.* [121]). Defects in the centromere, acting as the locus to which the spindle microtubules bind [122], would disrupt the process of correct segregation of chromosomes to each daughter cell during mitosis. 

With all of these functions taken together, it can be argued that the reason for the cell cycle arrest during the G1 phase is to reduce DNA damage by reducing oxidation, to repair DSB and to enhance centromere integrity. It is surprising then to find that the most reported function of PTEN—that of regulating the PI3K/AKT pathway—is not involved; in fact it has been reported that only cytoplasmic PIP3 is sensitive to PTEN [123]. However, it was found that enforced nuclear PTEN expression can reduce cellular levels of phosphorylated AKT [19].

### 3.4. DNA Damage

If ageing can be described as the increase in entropy of life sustaining systems, then that increasing entropy is due in large part to the accumulation of damage to DNA. In this report, one has seen that PTEN is a crucial factor regarding longevity. It can aid in caloric restriction, preventing an abundance of the sources of ROS from entering the cell; it enhances p53’s antioxidant capabilities, preventing the accumulation of ROS already in the cell; in a complex with p53 it helps repair DNA damage and through suppression of the PI3K/AKT and other pathways it prevents the emergence of tumors, malignant or otherwise. Through all these functions, PTEN promotes longevity. But one of, if not the key factor in longevity is DNA damage. Beyond the prevention of tumors, this prevents aberrant proteins and cells accumulating in tissues and further increasing the deterioration of the life sustaining systems. 

The deterioration within liver tissues due to accumulation DNA damage is a robust biomarker for ageing [124,125] and Ortega-Molina *et al.* found through immunofluorescence that PTEN transgenic mice showed a significantly reduced level of DNA damage in the liver compared to control mice. This effect was more pronounced the older the mice were. The older transgenic mice also performed significantly better during exercise tests, suggestive of good health. 

Whether through exogenous (radiation) or endogenous (ROS via metabolic processes) agents, cellular DNA is constantly under stress which can result in errors occurring during replication or mutations and other lesions. There are several DNA repair mechanisms which can effectively, in most cases, repair such lesions. These include nucleotide excision repair (NER), base excision repair (BER), mismatch repair (MMR), DNA double strand break repair (DSBR) and postreplication repair (PRR) [126,127,128]. The NER and BER pathways are most typically activated in response to damage done to individual bases. Single strand breaks (SSBs) and double strand breaks (DSBs) on the other hand require repair through more complex mechanisms such as homologous recombination (HR), non-homologous end joining (NHEJ) or single strand annealing (SSA) [129,130].

Of these, NER is the most versatile and flexible repair mechanism which has been conserved in most organisms, especially eukaryotes [131,132,133,134] and is crucially important in repairing lesions caused by UV [135,136]. This pathway involves proteins, such as downstream proteins of p53, which can detect, unwind and remove damaged DNA. There are two forms of NER: if the damage is linked to transcription there is transcription-coupled repair (TCR); if it is found to be linked to the genome in a general sense there is global genome NER (GG-NER). Linked to the NER pathways are the DNA damage response (DDR) pathways [137] which are activated to regulate cell cycle transitions, DNA repair and replication and apoptosis, processes which involve PTEN either indirectly or directly.

In studies using low suberythemal UV radiation, mice with down-regulated PTEN in epidermal cells showed a predisposition to skin tumors [13]. PTEN has been found to be significantly down-regulated in human skin malignant and premalignant lesions. As NER has been linked to UV based DNA damage repair, this strongly implicates PTEN in NER-related activation.

PTEN has also been indicated to positively regulate GG-NER activation through the promotion of XPC transcription in keratinocytes. XPC in turn is impaired via PTEN loss, crippling GG-NER. This occurs due to the AKT/p38 pathway which is critical for regulating XPC levels, through the increased nuclear translocation of the transcription repressor p130 [138]. Thus, PTEN positively regulates GG-NER through the suppression of AKT following DNA-damage [13]. 

As stated in previous sections, PTEN has a positive effect on DSBR through the up-regulation of Rad51. Various organisms studied with PTEN loss have shown evidence of DSBs and defective DSBR [72]. This has been disputed, however, by [139], who found that the initial phase of DNA damage sensing and modification that has been associated with DSBs is similar in cells with or without PTEN. As the authors used different cell lines than previous studies, this may suggest that PTEN’s role in DSBR is tissue specific.

Chk1 and Chk2, as well as regulating PTEN, play a significant role in DNA repair, activated by ATR [140], by activating p53 among other effectors [141,142].

### 3.5. Stem Cells

PTEN’s effect on stem cells originates from its influence on the regulation of cell growth and proliferation through the inhibition of PIP3. Three studies [81,143,144] found that PTEN deficiency in neuronal stem cells provided a strong proliferative response and promoted a greatly enhanced self-renewal capacity. This enhanced self-renewal capacity was found to be due to PTEN not being present to arrest the G_0_–G_1_ cell cycle and the decreased growth factor dependency of Pten null neural/stem progenitor cells. This was discovered through the deletion of PTEN in murine brains that led to macrocephaly (enlarged brains) and disturbing patterning of brain structures due to increased cell proliferation and decreased cell death. Follow up *ex vivo* experiments showed that PTEN loss dramatically increases total number of neurons in fetal brain and, more importantly, an increase in the number of neuronal stem cells capable of growth.

Zhang *et al.* found that deletion of PTEN in the murine hematopoietic system resulted in the depletion of current hematopoietic stem cells (HSCs) and increased the proliferation of leukemogenic stem cells [145]. The result of this was the mice developed myeloproliferative disorders which eventually led to leukemia. Yilmaz *et al.* in turn demonstrated that treating murine PTEN null cells with rapamycin, an mTOR inhibitor, blocked the growth of the leukemogenic stem cells and increased the proliferation of normal HSCs [146]. This strengthens the theory that PTEN’s effect on stem cells arises through its regulation of the PIP3/AKT pathway.

### 3.6. Senescence/Apoptosis

As PTEN has been shown to increase antioxidant activity, it seems counterintuitive that PTEN could promote senescence or apoptosis, especially when one considers that complete acute loss promoted a strong senescence response that opposes tumor progression [68], even though complete loss of PTEN has been found in many cancers, leading to speculation that loss of PTEN leads to tumorogenesis.

Be this as it may, Gil *et al.* showed that apoptotic stimuli promote the nuclear import of PTEN, implying the nuclear functions of PTEN include apoptosis [119]. While the mechanisms of PTEN’s pro-apoptotic functions are still unclear, PTEN has been found to augment doxorubicin-induced apoptosis in PTEN-null Ishikawa cells (cells that express truncated PTEN proteins) [147]. Of note in this study was that the doxorubicin reduced the levels of phospho-AKT/PKB suggesting PTEN role in apoptosis is through its regulation of the PIP3/AKT pathway. This hypothesis is supported by Vasudevan *et al.*’s, study that showed that NFκB, upregulated by AKT, suppresses PTEN activation which reduces apoptosis [34].

PTEN has also been found to induce apoptosis in a PI3K/AKT independent manner, through the association of p53. Much as PTEN and p53 work together to induce cell cycle arrest, it has been found that *p53*, in association with its family members *p63* and *p73*, can activate apoptotic genes in response to DNA-damage [148] and that a p73-PTEN complex enhances this response [149]. This at first appears to be in direct contrast to the above stated antioxidant activities of PTEN-p53 in response to DNA damage, but much as Wan *et al.* found that the level of doxorubicin affected the activation of PTEN induced apoptosis [147], the degree of DNA-damage may indicate whether PTEN-p53 would induce apoptosis or antioxidant release.

As the apoptotic functions of PTEN are controversial, it is difficult to ascertain the effect on longevity. Speculation, however, is that apoptosis effectively removes potentially harmful cellular toxins, misfolded proteins and damaged DNA from the system by destroying the cell [150,151]. This removes the potential for malignant cells or for these toxins to spread. While not a direct influence on longevity as has been seen elsewhere, this cellular housekeeping functions to keep tissues and organs healthy, indirectly increasing longevity. 

### 3.7. Caloric Restriction

A major breakthrough in the study of PTEN and how it relates to longevity has been the recent study done by Ortega-Molina [4]. Of special interest was not the link between tumor suppression and longevity as has been found with other tumor suppressors such as p53, Ink4a, Arf [124,152], but the effect PTEN has on caloric restriction and the implications this has for longevity. 

Ortega-Molina *et al.*’s study [4] showed both a median and mean increase in survival and longevity (between 9% and 16%). Testing showed this was due to the PTEN transgene, independent of other variables. It was observed that the PTEN transgene extended longevity independently of its tumor suppressive functions, as cancer-free mice also showed a significant increased longevity. This would leave its effects on the PI3K/AKT pathway, nuclear functions and/or insulin pathways to be the culprit.

The authors found that PTEN transgenic mice, both young and old, had lower fasting levels of glucose and insulin serum levels, and a significantly lower value of the insulin resistance index HOMA-IR compared with the wild type control mice. This observed effect has been a widely reported feature of long lived mice, namely that of a decreased insulin and insulin-like growth factor 1 (IGF1) signaling (IIS) pathway [153,154]. This comes to pass through negative feedback routes from the IIS pathway itself [77,89,155].

Ortega-Molina *et al.* further explored these negative feedback signals, especially in white adipose tissue (WAT) and found the main perpetrator was S6K which, along with mediating the nuclear export of PTEN as mentioned above, acts as a primary negative feedback signaler of the IIS pathway [156,157]. The WAT of transgenic PTEN mice presented reduced levels of AKT (as expected of increased PTEN activation) and also lower levels of phosphorylation in two relevant substrates of S6K, namely S6 and IRS1. S6K-mediated phosphorylation of IRS1 at Ser636/Ser639 is inhibitory for insulin signaling resulting in insulin resistance. Due to increased PTEN activation, the AKT pathway, which includes S6K, would be reduced, leading to less phosphorylation by S6K of IRS1 and thus, less insulin resistance. The authors further strengthened this hypothesis by feeding the mice, control and transgenic, a high-fat diet for six months (a well-established technique for inducing metabolic stress, insulin resistance and liver steatosis) and found that while the transgenic mice increased their body weight at similar levels to control mice, they were responsive to insulin injections while control mice were not. Control mice were also shown to have extensive liver steatosis while transgenic mice showed minimal or no signs.

Caloric restriction (CR) has been tested and observed in many organisms, including nematodes, mice and humans, and has been attributed to the effect of oxidation [158] from the IIS axis [15,159]. In *C. elegans* specifically, decreased PI3K (AGE-1) and increased PTEN (DAF-18) result in extended longevity [160,161,162] and in mice, reduced IIS activity also yields and extends longevity [4,154]. Studies show that CR results in decreased levels of IGF1, a potent stimulator of IRS1 and thus the PI3K pathway [163,164,165].

A commonly observed feature of long-lived mice, along with decreased IIS axis activity, is improved insulin sensitivity [153]. This is due to the earlier mentioned reduction in the negative feedback loop stemming from S6K [77,89,155]. Supporting this, the transgenic mice in Ortega-Molina’s study showed lower fasting levels of glucose and insulin serum levels and significantly lower value of the insulin resistance index HOMA-IR [4]. Serum levels of IGF1 were also significantly, if moderately, lower in transgenic mice than their controls.

Despite being hyperphagic, these transgenic mice had a decreased body weight compared to control mice (27%–28% decrease in young mice, 35%–44% decrease in old mice) [4]. Transgenic mice had a higher resting metabolic rate relative to lean mass, and this was shown to be an effect of PTEN independent of lean mass. Respiratory quotients and body temperature were ruled out as possible explanatory factors. Epididymal white adipose tissue (WAT) relative to body mass was also significantly lower in transgenic males, as well as serum levels of leptin and cholesterol. 

The brown adipose tissue (BAT) of transgenic mice also showed an increased activity. This is significant as BAT is an efficient source to dissipate heat [166,167], and the significant uptake of glucose in the BAT observed in the study suggests that the reduced weight of the transgenic mice may be due in part to their increased energy expenditure.

The importance of this to humans is twofold. Firstly, PTEN encourages calorie restriction which, through the decreased activity of the IIS axis and PI3K/AKT pathway, enhances longevity. Secondly, PTEN reduces insulin resistance, assists weight loss and lowers cholesterol, which when taken together means PTEN is crucial in the fight against diabetes, obesity and high blood pressure, again enhancing longevity.

## 4. Cancer Properties

There are two reasons that PTEN’s association with cancer is presented below. The first and most obvious reason is that cancer prevention is a straightforward extension of one’s longevity by preventing the possible mortality associated with cancer. While it has no effect on the genetic enhancement of longevity, it cannot be understated that prevention of an illness is key to extended longevity. 

The second reason for its inclusion in this report is that PTEN’s functions in relation to cancer are closely linked to its nucleic and cellular functions. Its inhibition of the PI3K/AKT pathway and its functions with p53 in DNA-damage are used for normally functional cells as well as often damaged and deteriorated cancer cells. It is by the inhibition of normal PTEN functions that malignant tumors may form.

### 4.1. Mutations

#### 4.1.1. Complete Loss/Haploinsufficiency

As with mutations that have a regulatory effect on the function of PTEN, mutations of PTEN is also a leading cause of cancer, with an estimated 50%–80% of sporadic tumors (includes endometrial carcinoma, glioblastoma and prostate cancer) and at 30%–50% in breast, colon, and lung tumors having monoallelic mutations of PTEN in common [1,109,168,169]. Complete loss of PTEN is associated with advanced cancers, metastases, and more recently been observed to be common in breast cancers caused by BRCA1 deficiency [170], and have been found mostly in endometrial cancer and glioblastoma [3].

Complete loss of PTEN is not certain to cause cancer, as it needs certain circumstances. Studies done on mice have shown that complete loss of PTEN is lethal in early development, yet heterozygous mice were viable only to develop a variety of tumors in later life [171]. More recently, however, a study showed that complete acute loss of PTEN did not induce hyperproliferation as expected, but promoted a strong p53-senescence response [68]. This suggests that complete loss of PTEN, in the absence of other mutations, may be detrimental to tumor growth, in contrast to the above finding of PTEN loss in various cancers. It does provide evidence though for why PTEN haploinsufficiency is more often presented in cancer than complete loss of allelic function.

The notion that complete loss of PTEN does not have a direct effect on cells, rather an indirect effect through downstream substrates is strengthened by several studies that have conditionally mutated both PTEN alleles using lox recombination to promote Cre recombinase specific tissues, both germ cells and somatic cells. The result was mutational inactivation of PTEN. This loss did not result in oncogenesis, rather subsequent generations of these PTEN null cells transformed into malignancies [81,144,172,173,174]. This suggests that, like the senescence response via p53, this oncogenic response is through another downstream effector of PTEN, possibly PIP3/AKT.

As reviewed in Salmena *et al.*, various mechanisms can create a gradual loss of PTEN and thus a gradation of tumor suppression, ranging from 0% loss to 50% heterozygous to 100% (homozygous) loss [20]. These mechanisms can include mutations, transcriptional repression, post-translation modification, epigenetic silencing and aberrant localization, many of which have been reviewed earlier in this report. Many of these, in combination, can create a continuum of PTEN functionality. For example, in mice PTEN is haploinsufficient for tumor suppression and thus 50% of total PTEN is insufficient for tumor suppression. 

Other studies using PTEN haploinsufficient mice have shown favorable tumor conditions where mice develop colonic adenomas, lymph node hyperplasia and prostate tumors at greater rates than controls due to deregulation of the PI3K/AKT pathway [16,175]. Other studies with mice have shown that loss of one PTEN allele promotes development of lethal polyclonal autoimmune disorders [176] and that PTEN heterozygosity is a driving force for epithelial cancers like prostate cancer [177], suggestive of PTEN haploinsufficiency. 

Despite this evidence, the theory that PTEN is haploinsufficient is still undetermined, although there are reports that substantiate this. However, PTEN is haploinsufficient for PHTS due to PTEN heterozygosity results in distinctive phenotypes such as developmental disorders and benign polyps; PHTS also severely increases the risk of developing malignant tumors [178]. Tumors that have been derived from individuals with Cowden syndrome have a tendency not to present detectable biallelic mutations of the *PTEN* gene [179], suggesting only one allele remains, strengthening the haploinsufficiency hypothesis.

#### 4.1.2. Germline Mutations

Germline mutations of PTEN have mostly been found in hereditary, autosomal dominant cancer syndromes with shared characteristics such as developmental defects and disorders, neurological deficits, multiple benign hamartomas and an increased risk of cancers. The aberrant growth and hamartomas have been shown to penetrate all three germ layers [180], evident of a mutation in the germline. 

These syndromes are Cowden syndrome, Lhermitte–Duclos disease, Bannayan–Riley–Ruvalcaba syndrome, and Proteus syndromes which collectively make up PTEN hamartoma tumor syndromes (PHTS) [92,181,182,183]. These have been associated with the deregulation of the PI3K/AKT pathway and PI3K inhibitors have been suggested as potential treatments [184]. Mice with PTEN mutations have been shown to be susceptible to tumors in various organs such as skin, prostate and mammary glands [13,185,186,187,188].

More recently, studies have reported *PTEN* germline mutations in patients with macrocephaly, mental retardation and autism spectrum disorders, while showing little to none of other PHTS symptoms [189]. These studies have shown that as many as 10% of cases involving autism spectrum disorders and macrocephaly had PTEN germline mutations [190]. While the connection between PTEN mutations and these neurological disorders remain unclear, much like PHTS, there have been associations made with the PI3K/AKT pathway.

#### 4.1.3. Somatic Mutations

Somatic mutations of PTEN have been documented in a variety of cancers and tumors, both at early and advanced stages. These mutations can lead to several different results: either inactivation of PTEN’s phosphatase activity through mutation on the *C*-terminal or partial or total loss of either mRNA and/or protein expression [191]. While typically a mutation is accompanied by loss of heterozygosity, resulting in the above mentioned possible haploinsufficiency, some tumors have appeared to evolve mechanisms to reduce concentration of PTEN without visible mutations of the gene [109].

Of note is that the frequency of PTEN mutation is nearly as great as that of p53 [171] and that both have been associated with total inactivation. Both have been putatively linked to stimulation of the PI3K/AKT pathway. With the closely linked activities of both genes in cancer suppression, cell cycle arrest and DNA-damage repair, it is unsurprising that both would be targeted for mutation. 

PTEN mutations have also been studied in model organisms such as Drosophila where it alters cell size, proliferation, apoptosis and cell migration [78,192,193], much like in humans. However, tissue-specific PTEN mutation in mice revealed that, among several phenotypes, PTEN inactivation did not result in tumors but created an environment that selects for tumor growth. This is potentially linked with the above mentioned Cre recombinase experiments.

### 4.2. Regulatory Effects Causing Cancer

Unsurprisingly, the regulatory effects of/on PTEN implicated in cancer development will have close ties with the previous section on regulation of PTEN. This is because, as PTEN is a tumor suppressor, its effects on tumors are only observed once it becomes inactive or down-regulated. While the earlier section focused on the mechanisms of regulation, this section will focus on the effects. 

Reports have indicated that a large part of PTEN’s tumor suppression is due to its nuclear functions. It was found that the absence of nuclear PTEN is commonly associated with the more aggressive diseases in patients with cutaneous melanoma [194,195], large B cell lymphoma [196], colorectal cancer [197], esophageal squamous cell carcinoma [198] and pancreatic islet cell tumors [112]. In these studies, the absence of nuclear PTEN is more markedly observed in undifferentiated and metastatic tumors [104]. With this, Liu *et al.* (2005) found that forced nuclear expression of PTEN antagonizes anchorage-independent growth [199]. 

A more general study regarding the tumor suppressive functions of PTEN was done by Ortega-Molina *et al.* [4]*.* Therein, the authors found that mice with an extra transgenic *PTEN* gene had significantly less incidence of fibrosarcomas than wild type mice. Histological exploration revealed a significantly reduced number of malignant tumors, most notably lymphomas and histiocytic sarcomas in transgenic mice.

While the exact mechanisms and causes are as of yet unclear, it can be suggested that the nuclear co-operation between PTEN and p53 in regards to DNA-damage may be significant. These two genes can arrest the cell cycle, leading to senescence and can initiated DSB repair of damaged DNA as well as other antioxidant effects through downstream effectors of p53. The antioxidant properties can prevent damage occurring to DNA; DNA repair mechanisms can repair any damage that does occur, and if these fail, the cell cycle can be arrested to induce senescence. All of those are working to prevent the formation of tumors.

Another oncogenic protein suppressed by nuclear function is the protein MSP58, which Okumura *et al.* discovered transformed PTEN null mouse embryonic fibroblasts, yet was abrogated in the presence of introduced PTEN [200,201]. MSP58 was also abrogated by phosphatase inactive PTEN mutant suggesting that, as with the other nuclear PTEN functions described earlier, this is done potentially through physical interaction of the *C*-terminal domain.

One of the most commonly observed methods by which PTEN has been suppressed has been during transcription. Methylation of the PTEN promoter region has been associated with reduced PTEN expression in various cancers [202]. The RAS-RAF-MEK-ERK pathway has been demonstrated to have a link with aberrant down-regulation of PTEN transcription in both epithelial and fibroblast cell types in human cancer cells through the factor c-Jun [33,203] and through transforming growth factor beta (TGFβ) dependent mechanism in pancreatic adenocarcinoma. miRNAs, and miR-21, in particular, disrupts expression of PTEN through binding to transcripted sequences [41,42].

As well as the nuclear functions regarding p53 described above, the suppression of PTEN would obviously lead to the unregulated behavior of the PI3K/AKT pathway leading to uncontrolled proliferation and cell growth. This would result in the proliferation of cells containing, potentially, deleterious segments of DNA, which may result in tumors.

There is also positive regulation of PTEN involved when regarding cancer. Tuberous sclerosis 1 and 2 (TSC1 and TSC2) forms a complex that inhibits mTOR and so inhibits S6K [204]. Along with reducing insulin resistance, this also reduces the nuclear export of PTEN. The TSC1 and 2 complexes, however, are down-regulated by AKTA via phosphorylation of TSC1. This phosphorylation leads to the degradation of the TSC1 and 2 complexes, disallowing it from inhibiting mTOR. Both TSC1 and TSC2 are tumor suppressors and are mutated in tuberous sclerosis (TSC). TSC is associated with benign hamartomas and brain abnormalities [119], much like PHTS, although both are required for efficient TSC suppression. As the TSC1 and 2 complex helps reduce incidence of PTEN nuclear export, and PTEN reduces AKT phosphorylation of the TSC1 and 2 complexes, one can see that the tumor suppressive functions of PTEN are much broader than only its own syndromes.

## 5. Conclusions

To sum up a lengthy report: PTEN has significant implications for extending human longevity through its actions on DNA-damage reduction, antioxidant activity, caloric restriction, inhibition of replication and tumor suppression. The importance cannot be overstated as PTEN overexpression can assist a variety of maladies including weight-related diseases such as diabetes to age-related diseases such as Alzheimer’s and Parkinson’s. Its function as a tumor suppressor can maintain an anguish-free life. It is because of this variety and necessity of function that PTEN is a vital subject for further research.

Through studies done on invertebrates [160,161,162] and on mammals [4] we have seen that the application of this knowledge is successful, that PTEN’s effect on longevity is not merely theoretical but practical. That PTEN can enhance longevity is no longer questionable, but neither is it irrefutable. Before any final concluding statements can be made, human trials with PTEN transfection must first be done. The authors of this study are currently working on cell culture trials, which is only the first step.

PTEN alone cannot extend longevity indefinitely, however, Ortega-Molina *et al.* [4] found only a 9%–16% increase in longevity, and while this is a significant milestone, this is hardly the fountain of youth that Ponce de León dreamt of. This is not to say that such a dream may not happen, merely that PTEN alone would not accomplish it. Jaskelioff *et al.* presented findings that telomerase can reverse tissue damage in aged mice [14]. This rejuvenative quality bodes well as a potential partner for PTEN, and its most important feature, that of telomere extension, could potentially extend longevity as long as needed. PTEN is well suited as a partner for telomerase due to its tumor suppressive quality. This is because of two reasons. Telomerase have been commonly associated with cancer and a tumor suppressor may prevent this. However, more importantly, the longer one lives, the probability of having cancer increases. It is PTEN’s tumor suppressive quality that sets it apart from other recent studied genes such as *SIRT1*.

The variety of genes, proteins and enzymes being studied today show how the interconnectivity of the human system also necessitates a complex solution to longevity. Whether this is achieved through the main pathways of telomerase, SIRT1, PTEN or others remains to be seen. What must be done now is testing, and further testing, until an answer is found. With the importance of such work, it deserves no less. While human trials oblige a lengthy testing time, it is an inevitable obstacle that must be overcome if Pons de León’s dream is to be fulfilled.
